# Flow-Induced Structuring of Polypropylene-Based Composites for Improved Mechanical and Thermal Performance

**DOI:** 10.3390/polym18202588

**Published:** 2026-10-20

**Authors:** Rossella Arrigo, Mohammad Parhami Alamdari, Alberto Frache

**Affiliations:** 1 Department of Applied Science and Technology, Politecnico di Torino, Viale Teresa Michel, 5, 15121 Alessandria, Italy; mohammad.parhamialamdari@studenti.polito.it (M.P.A.); alberto.frache@polito.it (A.F.); 2 Consorzio Interuniversitario Nazionale per la Scienza e Tecnologia dei Materiali (INSTM), Via G. Giusti 9, 50121 Florence, Italy

**Keywords:** non-isothermal elongational flow, particle alignment, thermal conductivity, fiber drawing, uniaxial stretching

## Abstract

Polypropylene (PP)/hexagonal boron nitride (BN) composites were formulated through melt compounding and subsequently subjected to non-isothermal uniaxial elongational flow to assess how flow-induced microstructural evolution governs mechanical and thermal performance. Rheological characterization of the isotropic, as-compounded materials revealed increasing melt elasticity with BN loading. At 25 wt% BN, a pseudo-solid-like response emerged, suggesting the development of an incipient interconnected filler structure. In the isotropic state, BN increased stiffness, tensile strength, and thermal conductivity, with only minor effects on PP crystallinity. The application of the non-isothermal elongational flow further improved the mechanical response, particularly at high BN loading, indicating an additional contribution from flow-induced reorganization of the filler phase. X-ray diffraction confirmed the preferential alignment of BN platelets along the fiber direction. This structural reorganization was accompanied by an increase in thermal conductivity of approximately 56% for PP containing 25 wt% BN. Overall, the obtained results demonstrate that non-isothermal elongational flow can effectively tailor BN orientation and polymer-chain organization, providing a solvent-free and scalable route to enhance the mechanical and thermal performance of PP/BN composites.

## 1. Introduction

The continued miniaturization and increasing integration density of electronic devices, combined with rising power levels, have led to considerably higher heat generation in ever more constrained volumes [1,2,3,4]. Inadequate thermal dissipation promotes heat accumulation, thereby accelerating degradation processes and limiting long-term operational stability [5]. As a result, the development of effective thermal management strategies has become essential for ensuring stable and long-term device operation [6,7]. In recent years, polymeric materials have been commonly applied in electronics, automotive, and aerospace fields due to their lightweight, corrosion resistance, mechanical flexibility, low cost, and ease of processing [8,9]. Among the thermoplastics, polypropylene (PP) is one of the most common materials used because of its good balance of chemical stability, mechanical properties, costs, and processability in the molten state [10,11]. Despite its advantages, it is observed that thermoplastics like PP are normally characterized by inherently low thermal conductivity (~0.1–0.3 W m^−1^ K^−1^), making it unsuitable for direct use in heat-spreading or heat-removal components [12]. An effective and viable approach to overcome the low thermal conductivity of polymers is to incorporate thermally conductive fillers or nanofillers within polymeric matrices [13,14]. Ceramic fillers are particularly beneficial for polymer composites or nanocomposites because they can enhance thermal conductivity while maintaining electrical insulation [15,16,17]. In this context, hexagonal boron nitride (h-BN) has been widely investigated as a filler due to its high intrinsic thermal conductivity, excellent dielectric properties, chemical stability, and layered two-dimensional (2D) structure [18,19,20]. Some researchers have adopted simple direct mixing of BN with polymers; however, significant improvements in thermal conductivity often require very high filler loadings (>50% (volume)), which may adversely affect mechanical properties [21]. In addition to the intrinsic properties of BN, the thermal performance of BN/polymer composites is strongly governed by platelet geometry, orientation, and interfacial characteristics [22].

Regarding orientation, multiple alignment strategies have been reported for polymer composites, including doctor-blade-assisted film fabrication [23], magnetic- [24] and electric-field-assisted [25] methods, oscillatory shear [26], electrospinning-based [27], tape-casting [28], and freeze-casting followed by sintering and polymer infiltration [29] approaches. As an example, Yuan et al. [30] indicated that the application of magnetic field assistance to the alignment of the h-BN platelets resulted in a 44.5% enhancement in the thermal conductivity of the composites when the platelets were aligned parallel to the heat flow direction compared to the random orientation case. In a similar study, Shen et al. [23] demonstrated that surface modification of the h-BN platelets combined with the doctor blading-induced alignment of the particles can lead to materials with improved thermal transport properties. In fact, the thermal conductivity of the composites was found to be about 5.4 W m^−1^ K^−1^ at a BN loading of 10 vol% for the aligned composites, much higher than that of the material containing randomly dispersed h-BN platelets. Even though there are practical challenges in achieving precise platelet alignment in polymer matrices, these findings highlight that orientation control is an effective strategy to improve thermal conductivity without relying solely on extremely high filler loadings.

In addition to the alignment of the platelets, the exfoliation of the BN filler to thinner boron nitride nanosheets (BNNS) has been a main area of research in the improvement of the thermal conductivity of the composites [31]. There are two major preparation strategies for BNNS, namely the “top-down” and “bottom-up” methods [32]. On the one hand, in the top-down approach, stacked BN layers are separated by overcoming interlayer interactions using processes such as mechanical stripping [33,34], alkali hydrothermal-assisted mechanical high-shear exfoliation [35], micro-fluidization [36], liquid-metal-assisted exfoliation [37], ultrasonic stripping [38,39], and ball milling [40], etc. For instance, Lee et al. [41] demonstrated that hydroxide-assisted ball milling enabled the exfoliation of bulk h-BN into hydroxyl-functionalized BN nanoplatelets with an average lateral size of approximately 1.5 µm and a yield of 18%, while preserving the in-plane structure to a large extent. As another example, Fu et al. [42] developed a molten hydroxide-assisted liquid exfoliation method to produce hydroxylated boron nitride nanosheets (HBNNS). The resulting layered heat spreader indicated a high in-plane thermal conductivity of 51.1 W m^−1^ K^−1^, which could be further increased by about 14% after annealing, while maintaining excellent thermal stability up to 700 °C. On the other hand, the bottom-up approach involves the direct growth of BNNS through techniques, most commonly including chemical vapor deposition [43,44], physical vapor deposition [45], surface polarization [46], and template-assisted [47] routes. A representative example is the work of Kim et al. [48], who reported the growth of large-area monolayer h-BN on Cu foil using low-pressure chemical vapor deposition, with continuous monolayer films forming after growth times between 10 and 120 min. Although bottom-up approaches may provide BNNS with high structural quality, they are often associated with high costs and relatively low production yield. Consequently, their large-scale application remains limited. Therefore, it is more common for top-down methods to be used for thermally conductive polymer composites due to their scalability and cost-effectiveness.

One of the major challenges in processing polymer-based nanocomposites is the tendency of nanofillers to agglomerate. This leads to nonuniform filler dispersion and can negatively affect the properties of the resulting materials [49,50,51]. Compatibilizers are often incorporated to improve compatibility between nonpolar polymer matrices and nanofillers and promote intercalation [52,53]. These chemically assisted approaches can improve filler dispersion and composite properties. However, their cost, processing complexity, and scalability may limit their practical application.

Melt processing involving elongational flow and stretching under non-isothermal conditions offers a practical and industrially feasible approach to improving nanofiller dispersion. These methods are solvent-free, scalable, and compatible with conventional polymer-processing technologies [54,55,56,57]. Elongational flow involves stretching that can be uniaxial or biaxial [58,59,60]. Such flow fields occur in several industrial operations, including fiber spinning, film blowing, blow molding, and stretching processes. In these operations, the polymer melt undergoes extensional deformation during shaping [61,62,63].

Compared with conventional shear-dominated processing, elongational flow can generate stronger extensional stresses. These stresses can promote the progressive breakup of filler aggregates and agglomerates formed during melt compounding, resulting in more uniform dispersion within the polymer matrix. Elongational deformation can also induce preferential alignment of anisotropic fillers along the stretching direction. This produces anisotropic microstructures and direction-dependent properties. Such orientation effects are particularly relevant for layered fillers, whose alignment can substantially influence the transport and mechanical performance of the composites [64,65].

More broadly, elongational flow can deform and reorganize dispersed phases within multiphase polymer systems. Significant morphological changes have been reported in polymer blends [66,67] and micro- and nanocomposites [68,69]. In systems containing layered, anisotropic fillers such as BN [65], these changes can include improved dispersion and preferential platelet alignment. In some cases, elongational flow may also facilitate the development of more highly exfoliated structures.

A representative example is provided by He et al. [70], who used a self-designed eccentric rotor extruder to continuously prepare oriented UHMWPE/BN composites. Elongational flow promoted the alignment of BN sheets and intimate contact between them. The resulting thermal conductivity reached 3.75 W/mK, exceeding that of materials prepared by conventional torque mixing. More recently, Sun et al. [71] investigated high-speed uniaxial stretching of melt-processed PE/BN nanocomposites. This process promoted the orientation and redistribution of BN nanosheets within the polymer matrix. At 40 wt% BN, the resulting materials exhibited an in-plane thermal conductivity of 6.56 W/mK, a tensile strength above 90 MPa, and a modulus above 3 GPa.

Herein, PP-based nanocomposites containing 5, 15, and 25 wt% BN were prepared to investigate the role of non-isothermal elongational flow in tailoring the morphology and properties of BN-filled PP systems. BN particles were first incorporated into the PP matrix by melt compounding, and the rheological, morphological, and thermal behavior of the corresponding isotropic materials was evaluated. The effect of subsequent elongational deformation was then assessed in terms of microstructural evolution, filler dispersion, platelet orientation, and the resulting thermal and mechanical performance. Overall, this study explores a solvent-free, scalable, and industrially relevant route for improving PP/BN composites without preliminary chemical modification of the filler.

## 2. Materials and Methods

### 2.1. Materials

Polypropylene Moplen HP500N (melt flow rate: 12 g/10 min at 230 °C/2.16 kg; density: 0.90 g cm^−3^), supplied by LyondellBasell (Rotterdam, The Netherlands), was used as the polymer matrix.

Hexagonal boron nitride (average particle size ~1 μm, density 2.29 g/mL at 25 °C) from Sigma Aldrich (Milano, Italy) was employed as a nanofiller.

### 2.2. Processing

PP-based composites containing 5 (PP+5BN), 15 (PP+15BN), and 25 wt% BN (PP+25BN) were prepared by melt compounding in a ThermoFisher (Waltham, MA, USA) Process 11 co-rotating twin-screw extruder (feed rate: 100 g/h; screw speed: 70 rpm; flat temperature profile: 190 °C).

The melt-compounded materials were subsequently processed under non-isothermal elongational flow using a RheoSpin apparatus (IdeaInstr, Termini Imerese, Italy). In this setup, the molten extrudate exiting the extruder is subjected to uniaxial stretching by a system of pulleys that captures the hot filament and transports it to a final pulley operating at constant speed or acceleration. Because the filament is stretched under non-isothermal conditions, it progressively cools during elongation until solidification. This approach enables fibers to be collected at different draw ratios, calculated using Equation (1):

(1) $$\mathrm{DR}\;=\;{\mathrm{diameter}}_{\mathrm{extrudate}}^{2}/{\mathrm{diameter}}_{\mathrm{fiber}}^{2},$$

Unfilled PP was processed under the same conditions, with all processing parameters kept unchanged.

### 2.3. Characterizations

Rheological analyses were carried out using an ARES (TA Instruments, New Castle, DE, USA) parallel plate rheometer with a plate diameter equal to 25 mm. Frequency sweep measurements were performed at 190 °C, within a frequency range of 0.01–100 rad/s, in the linear viscoelastic region of each material (preliminarily determined through strain sweep measurements at the same temperature). Rheological tests were performed on disc-shaped samples (diameter: 25 mm, thickness: 1 mm) obtained through compression molding, using a Collin (Maitenbeth, Germany) P200T hot plate press, operating at 190 °C, 100 bar, for 5 min.

The morphology of the melt-compounded composites and fibers was investigated using a Zeiss (New Castle, DE, USA) EVO 15 scanning electron microscope (SEM) operated at 20 kV and a working distance of 8.5 mm. Samples were cryo-fractured in liquid nitrogen and sputter-coated with gold before observation.

Differential scanning calorimetry (DSC) analyses were performed using a DSC Q20 (TA Instruments, New Castle, DE, USA) under nitrogen. The thermal cycle consisted of a first heating scan from −50 to 220 °C, cooling from 220 to 50 °C, and a second heating scan from −50 to 220 °C. A heating/cooling rate of 10 °C min^−1^ was used in all steps. The crystallinity degree of PP was calculated using Equation (2):

(2) $${Χ}_{c}\;=\;\frac{{∆H}_{m}}{{∆H}_{m,\;100\%}\;\times \;(1\;-\;x)}\;\times \;100,$$

where ∆*H_m_* is the melting enthalpy of the sample, ∆*H_m_*_, 100%_ is the melting enthalpy of a 100% crystalline PP (207 J/g [72]), and *x* is the BN content.

Mechanical characterization of the isotropic samples and fibers was performed at room temperature using an Instron^®^ (Norwood, MA, USA) 5966 universal testing machine equipped with a 2 kN load cell and a crosshead speed of 100 mm/min. Isotropic dog-bone specimens (ISO 527-2 type 5A [73]) were prepared by compression molding with a Collin P 200T press at 190 °C and 100 bar for 5 min. At least five specimens were tested for each material and, for the fibers, for each DR value; the reported results in terms of elastic modulus (E), tensile strength (TS), and elongation at break (EB) are average values.

X-ray diffraction (XRD) analyses were performed on both isotropic samples and fibers using a PANalytical (Malvern, UK) X’Pert PRO diffractometer with Cu Kα radiation (λ = 1.540562 Å) and a scanning step of 0.026°.

Thermal conductivity was evaluated by the transient plane source method according to ISO 22007-2 [74] using a TPS 2500S instrument (Hot Disk AB, Göteborg, Sweden) equipped with a Kapton sensor (radius: 3.189 mm). Measurements were performed at 23.00 ± 0.01 °C using a silicone-oil bath (Haake A40) coupled with a Haake AC200 temperature controller (Thermo Scientific, Waltham, MA, USA). Concerning the anisotropic specimens, multiple fibers were aligned in the same direction and compacted using a compression press at low temperature to minimize inter-fiber voids and obtain mechanically coherent specimens while preserving the morphology induced by elongational flow.

## 3. Results and Discussion

### 3.1. Isotropic (As-Compounded) Materials

Figure 1A shows the complex viscosity as a function of frequency for neat PP and the PP/BN composites. As expected, unfilled PP exhibits a non-Newtonian response, with a mild shear-thinning region at high frequencies. For both PP+5BN and PP+15BN, the complex viscosity curves retain an overall shape and frequency dependence comparable to those of neat PP, although they are progressively shifted toward higher values as the BN content increases. Therefore, at these filler concentrations, BN mainly produces a quantitative increase in melt viscosity without substantially altering the rheological behavior of the PP matrix. A distinctly different response is observed for PP+25BN, which exhibits a much larger increase in complex viscosity, particularly in the low-frequency region with the appearance of an apparent yield stress, together with more pronounced shear-thinning behavior. These modifications in the material rheological response suggest that, at the highest BN content, filler–filler interactions become increasingly relevant owing to the shorter interparticle distances and begin to affect the long-time relaxation dynamics of the composite.

The storage-modulus curves reported in Figure 1B support this interpretation. The addition of 5 and 15 wt% BN progressively increases the modulus, but the corresponding curves retain a frequency dependence similar to that of the unfilled matrix. Thus, although these composites exhibit a more pronounced elastic response, their rheological behavior remains predominantly matrix-controlled. In contrast, PP+25BN shows a marked deviation from the response of neat PP. In particular, its storage modulus tends toward a plateau in the low-frequency region, indicating a substantial suppression of the terminal relaxation of the PP chains and the onset of a pseudo-solid-like response. This qualitative change can be associated with the development of an incipient interconnected BN structure, which increasingly governs the long-timescale response of the material. This filler structure can sustain part of the applied stress over long timescales, thereby maintaining an appreciable elastic contribution even at low frequencies. Similar weakly frequency-dependent storage moduli and pseudo-solid-like responses have been reported for polymer/layered-silicate nanocomposites [75,76] and BN-containing polymer composites [77].

The stress-relaxation results reported in Figure 2 further support this interpretation. In fact, neat PP and the composites containing 5 and 15 wt% BN exhibit qualitatively similar relaxation behavior. Although their initial modulus values differ, G(t) decreases continuously with time and approaches very low values at long relaxation times. Therefore, the addition of 5 and 15 wt% BN does not substantially alter the relaxation kinetics of the PP matrix, indicating that the polymer chains are still able to recover an almost fully relaxed configuration after the application of strain.

Once again, a markedly different behavior is observed for PP+25BN. In fact, this material exhibits a considerably slower modulus decay and retains much higher G(t) values throughout the investigated time range. In particular, at long relaxation times, the modulus tends toward a non-zero residual value rather than approaching the values measured for neat PP. The observed response indicates that the long-range relaxation of the PP macromolecules is strongly hindered at the highest BN concentration, leading to the onset of pseudo-solid-like behavior. The altered relaxation kinetics can be attributed to the reduced distance between the BN platelets and the consequent formation of an interconnected filler structure that restricts the long-range motion of the polymer chains. The slow rearrangement of this proposed filler structure can therefore account for the incomplete stress relaxation observed within the experimental time window. Such an association between filler-mediated connectivity and delayed relaxation has also been reported in other nanofilled polymers [78]. These results are fully consistent with the low-frequency plateau observed in the storage-modulus curve of PP+25BN, confirming that a qualitative modification of the rheological response occurs only at the highest investigated filler content.

In order to assess the morphology established after the melt compounding step, SEM observations were performed on the cryo-fractured surfaces of the BN-containing materials and representative micrographs of the composites with the lowest and highest BN contents are shown in Figure 3. It can be clearly observed that BN is generally well dispersed and distributed throughout the PP matrix at both filler contents, indicating that melt compounding provides satisfactory incorporation of the platelets. The filler is mainly visible as fine platelet-like features embedded in the fractured matrix, although a limited number of BN-rich domains and residual agglomerates are also present. Interestingly, discrete agglomerates are more readily distinguished in PP+5BN, where isolated filler-rich regions stand out against the surrounding PP matrix. In PP+25BN, the fracture surface contains a much larger and more uniformly distributed population of BN platelets. The shorter interparticle distances make the filler distribution appear more continuous, and isolated agglomerates are therefore less evident, although some local particle-rich regions remain. Similar effects of filler size, loading, and mixing conditions on BN dispersion and aggregation in polymer matrices have been reported previously [19,20,79,80]. Overall, the morphological analysis indicates a satisfactory initial distribution of BN in both isotropic composites, with limited residual agglomeration that is more visually pronounced at the lower filler content.

Table 1 summarizes the melting temperature, crystallinity degree, tensile properties, and thermal conductivity of the isotropic materials. The melting temperature is essentially unaffected by BN addition, remaining within the narrow range of 165.9–166.3 °C. Similarly, the PP crystallinity is only slightly affected by BN addition, showing a modest upward trend with increasing filler content. In contrast, the tensile response is markedly affected by the filler content. The elastic modulus increases progressively with BN loading, reaching an enhancement of approximately 94% for PP+25BN compared with neat PP. Tensile strength follows a similar increasing trend, whereas elongation at break progressively decreases as the filler content rises. Thus, the rigid BN platelets reinforce the PP matrix and restrict segmental deformation, increasing stiffness and strength at the expense of ductility. The increase in tensile strength further suggests that the generally satisfactory filler dispersion observed by SEM allows effective stress transfer, while the reduction in elongation is consistent with the progressive constraint imposed on matrix deformation by the growing solid fraction [65,79,81]. Thermal conductivity also increases monotonically with BN content, with PP+25BN reaching a value nearly twice that of neat PP. This progressive enhancement is attributed to the increasing fraction of the highly thermally conductive BN phase and to the reduced separation between adjacent platelets, which promotes the formation of more effective heat-conduction pathways [19,20,79,80,81].

### 3.2. Anisotropic Materials (Fibers)

To evaluate the effect of non-isothermal elongational flow on the mechanical response of the materials, the fibers collected at different draw ratios (obtained by subjecting the molten extrudates to uniaxial stretching) were characterized through tensile tests. The obtained results, in terms of variation in the dimensionless elastic modulus and dimensionless tensile strength as a function of DR, are depicted in Figure 4A,B. The dimensionless properties were calculated as the ratios between the value measured for a fiber produced at a given draw ratio and the corresponding value measured for the as-compounded material. This representation allows for removing the differences associated with the initial mechanical properties of the isotropic materials and directly highlights the relative reinforcement produced by elongational flow [82].

As shown in Figure 4A,B, both the dimensionless elastic modulus and dimensionless tensile strength generally increase with draw ratio. The enhancement observed for neat PP can be associated with the progressive orientation and extension of the polymer chains along the stretching direction. PP+5BN exhibits trends that are very similar to those of unfilled PP: despite the higher mechanical properties of the corresponding isotropic composite, the relative improvement produced by drawing remains comparable to that observed for the unfilled matrix. This result indicates that, at the lowest BN content, the increase in modulus and tensile strength with draw ratio is mainly governed by the orientation of the PP chains, while the contribution arising from a possible evolution of the filler morphology is limited. PP+15BN shows an intermediate behavior, with a more pronounced increase in both dimensionless properties, especially at high draw ratios. The strongest dependence on draw ratio is observed for PP+25BN, for which both tensile properties increase much more rapidly than for neat PP and the lower-filled composites. Therefore, at high BN contents, polymer-chain orientation alone cannot fully account for the observed reinforcement, and additional phenomena involving the embedded BN particles need to be considered for explaining the mechanical response of the fibers [82,83,84].

To separate the contribution of polymer-chain orientation from that associated with the possible evolution of BN organization, the dimensionless values of the composites were further normalized with respect to the corresponding dimensionless values of neat PP at the same draw ratio. Through this normalization, a nearly constant value close to unity indicates that the enhancement of the mechanical property is primarily attributable to the orientation of the PP chains. Conversely, an increasing normalized value indicates an additional reinforcing contribution related to the evolution of the filler morphology during stretching [82,83].

The normalized results reported in Figure 5 clearly distinguish the behavior of PP+5BN from that of PP+25BN. More specifically, for PP+5BN, both normalized elastic modulus and tensile strength remain approximately constant and close to unity for all investigated draw ratios. Thus, the mechanical enhancement of these fibers can be mainly ascribed to polymer-chain orientation, without evidence of a substantial additional contribution from the BN particles. In contrast, both normalized quantities progressively increase with draw ratio for PP+25BN. This behavior suggests that elongational flow induces a gradual evolution of the composite microstructure in addition to the orientation of the polymer matrix, which contributes to the mechanical reinforcement [83,84].

The morphological characterization performed by SEM further supports the interpretation derived from the tensile behavior of the fibers. As shown in Figure 6, several BN-rich domains and residual agglomerates are still visible in PP+5BN, and the overall morphology remains relatively similar to that observed for the corresponding as-compounded material. Conversely, the anisotropic PP+25BN system exhibits a markedly more homogeneous morphology than its isotropic counterpart, with the micron-scale agglomerates observed before stretching no longer clearly distinguishable. In addition, the BN platelets display a pronounced preferential orientation along the stretching direction. These observations indicate that, at the highest filler content, non-isothermal elongational flow induces a substantial reorganization of the BN phase, involving preferential alignment of the anisotropic platelets along the fiber axis, partial disruption of the pre-existing filler agglomerates, and improved particle dispersion and distribution within the PP matrix. This flow-induced morphological evolution is consistent with the tensile behavior discussed above. In particular, the disruption of the agglomerates and the resulting improvement in filler dispersion increase the effective polymer–filler interfacial area, while the preferential platelet orientation promotes a more efficient load transfer along the fiber axis. The combined effects account for the additional reinforcement observed for PP+25BN fibers at increasing draw ratios, beyond the contribution associated with polymer-chain orientation alone.

The preferential orientation of the BN particles in the PP+25BN system resulting from the application of the elongational flow was further demonstrated through XRD analyses. In particular, Figure 7 compares the XRD patterns of the as-compounded PP+25BN composite and the corresponding fiber obtained under elongational flow. Both patterns show the characteristic reflections of hexagonal boron nitride, namely the intense (002) basal reflection at approximately 2θ = 27°, the (100) reflection at approximately 41°, and the higher-order (004) reflection at approximately 55°. The absence of appreciable shifts in the positions of these peaks indicates that elongational processing does not significantly modify the crystalline structure or interlayer spacing of BN. The main differences between the two samples instead concern the relative intensities of the BN reflections, which are sensitive to platelet orientation.

As already reported in the literature [24,30], in the adopted reflection geometry, the (002) and (004) reflections are mainly associated with platelets whose basal planes are preferentially oriented parallel to the investigated sample surface, whereas the relative intensity of the (100) reflection increases when a larger fraction of platelets is arranged in an edge-on or through-plane configuration. The isotropic PP+25BN sample exhibits clearly detectable (002), (100), and (004) signals, consistent with a comparatively broad distribution of platelet orientations. After elongational drawing, the (002) reflection becomes strongly predominant, whereas the (100) reflection is markedly reduced and is no longer clearly distinguishable. This evolution of the relative peak intensities provides evidence of a preferential orientation of the BN platelets induced by elongational flow. In the adopted measurement configuration, this result indicates that the platelet basal planes become preferentially aligned parallel to the fiber surface and, consistently with the drawing geometry, along the fiber axis.

Finally, Figure 8 compares the thermal conductivity of unfilled PP and PP+25BN before and after the application of the elongational flow. For the neat matrix, the drawing induces only a moderate increase in thermal conductivity with respect to the as-compounded material, likely ascribable to the preferential orientation of the polymer chains promoted by the uniaxial stretching. A more significant enhancement is observed for PP+25BN, whose thermal conductivity increases from 0.52 W/mK in the isotropic material to approximately 0.82 W/mK in the fiber. The considerably larger improvement observed for the composite indicates that the enhancement cannot be attributed solely to PP-chain orientation, but also involves a flow-induced reorganization of the BN phase. This interpretation is consistent with the XRD results in Figure 7, which show that elongational flow induces a preferential orientation of the BN platelets, becoming predominantly aligned with the fiber surface and stretching direction. Such alignment is particularly favorable for thermal transport because it allows the highly anisotropic in-plane conductivity of BN to be more effectively exploited, while the associated redistribution and closer spatial arrangement of the platelets can generate less tortuous and more continuous heat-conduction pathways, reducing the amount of poorly conductive polymer separating adjacent particles [19,20,22]. Similar effects have been reported for BN-containing polymer composites processed under elongational or uniaxial flow. He et al. [70] attributed the enhanced thermal conductivity of BN-containing composites to the improved orientation and intimate contact between BN sheets, whereas Sun et al. [71] showed that high-speed melt stretching promotes the orientation and redistribution of BN nanosheets, resulting in strongly enhanced in-plane thermal transport. A comparable structure–property relationship has also recently been demonstrated for fibers assembled from two-dimensional graphene sheets: increasing the draw ratio progressively improved sheet alignment and structural compactness and was accompanied by a substantial increase in thermal conductivity [85].

## 4. Conclusions

In this work, PP/BN composites containing up to 25 wt% BN were formulated and characterized, aiming to evaluate the possible effect of non-isothermal elongational flow on the material morphology and, consequently, on mechanical and thermal performance. The obtained results showed that, in the isotropic melt-compounded samples, increasing BN content progressively enhanced stiffness, tensile strength, and thermal conductivity, while producing only minor changes in the melting behavior and crystallinity of the PP matrix. Furthermore, the rheological analyses demonstrated the onset of an interconnected filler structure and a transition toward pseudo-solid-like behavior for the material containing the highest amount of BN. The application of non-isothermal elongational flow induced effects that extended beyond the orientation of the PP chains. At low BN content, the improvement in the mechanical properties of the fibers was mainly governed by matrix orientation. Conversely, for PP+25BN, the uniaxial stretching promoted a substantial evolution of the composite morphology, involving the partial disruption of the filler agglomerates and the preferential alignment of the anisotropic BN platelets along the fiber direction. This flow-induced reorganization improved filler dispersion and stress transfer across the polymer–filler interface, leading to larger increases in both stiffness and tensile strength with draw ratio. At the same time, the particle alignment promoted the achievement of a significant increment in the thermal conductivity for the material containing 25 wt% BN, whereas unfilled PP exhibited only a modest increase. Overall, the obtained results demonstrate that controlling elongational deformation during processing can improve both mechanical reinforcement and thermal conductivity without relying on chemical modification of BN. The approach therefore represents a simple, solvent-free, and potentially scalable strategy for producing thermally conductive PP-based composites with tunable anisotropic properties.

## Figures and Tables

**Figure 1:**
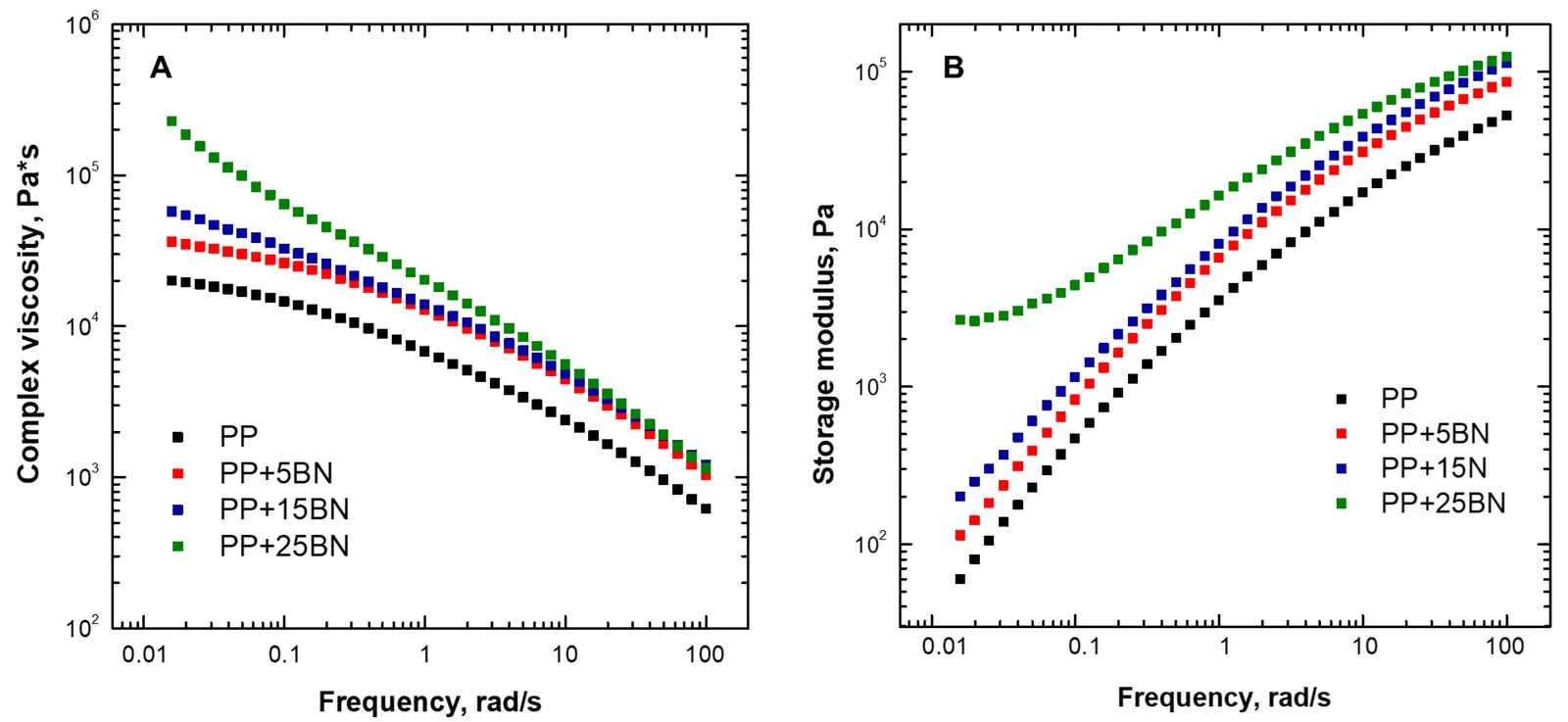
Complex viscosity (**A**) and storage modulus (**B**) curves as a function of frequency for all investigated samples.

**Figure 2:**
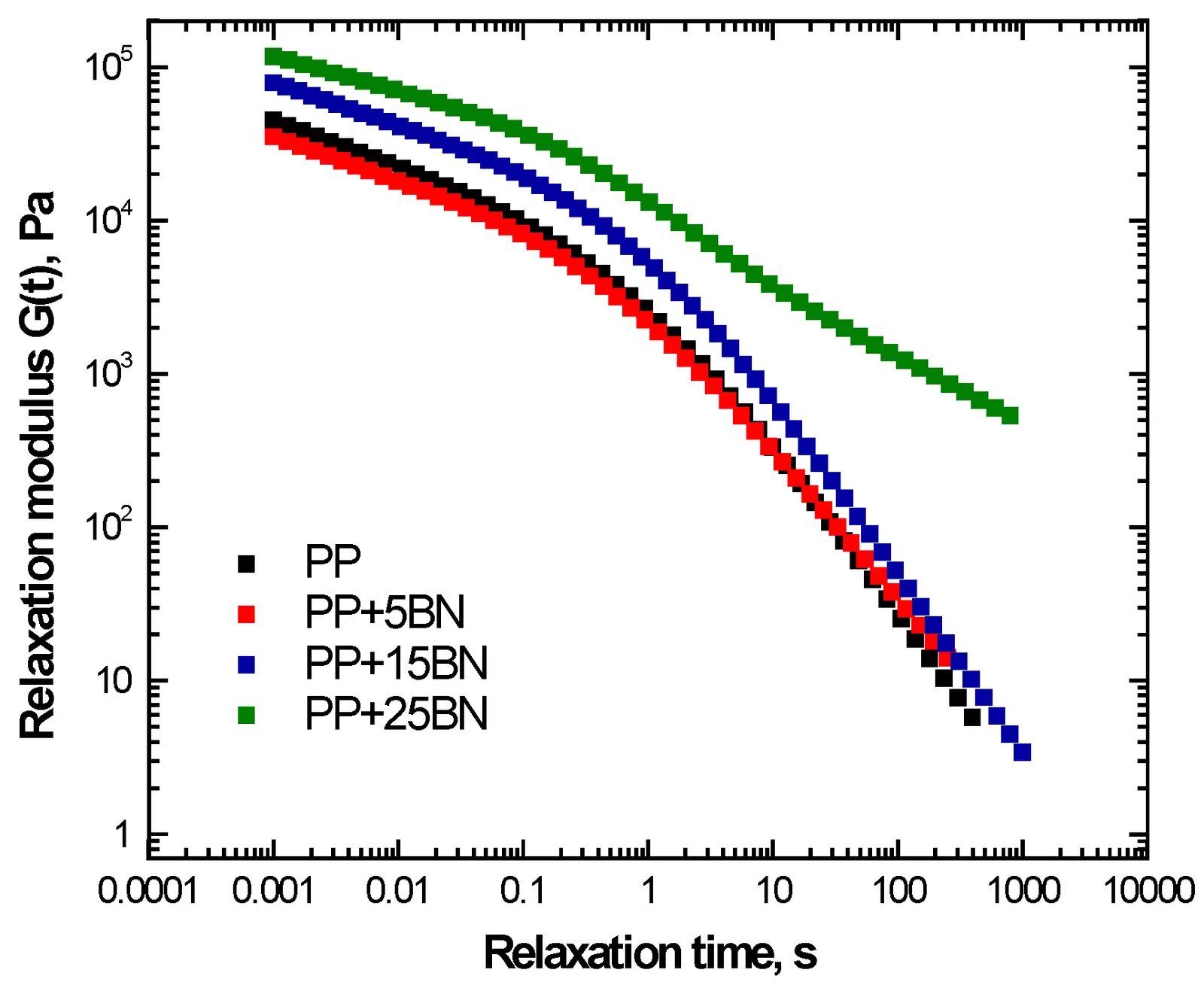
Stress relaxation curves for all investigated materials.

**Figure 3:**
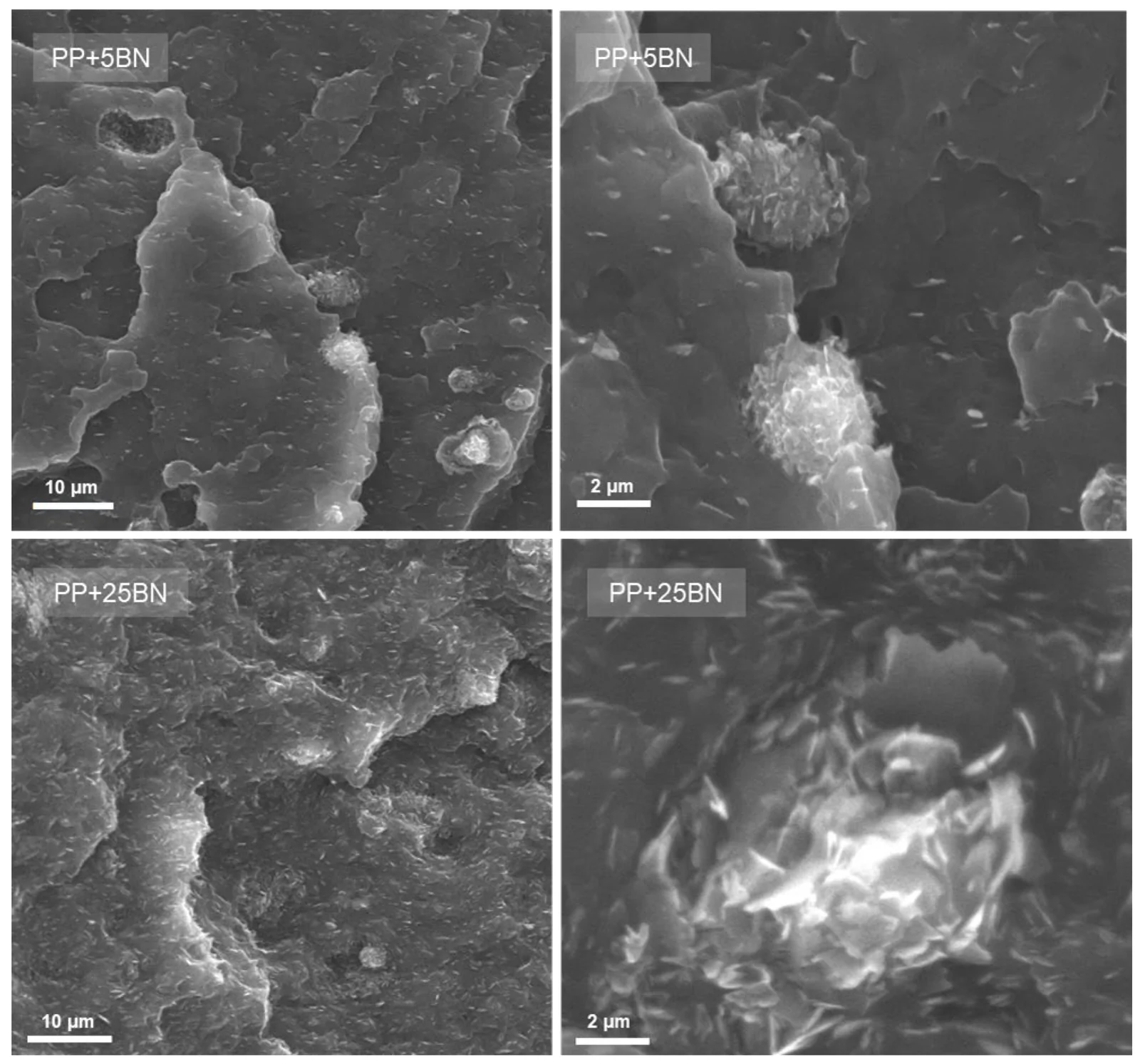
SEM micrographs of melt-compounded (isotropic) PP composites containing 5 and 25 wt% BN.

**Figure 4:**
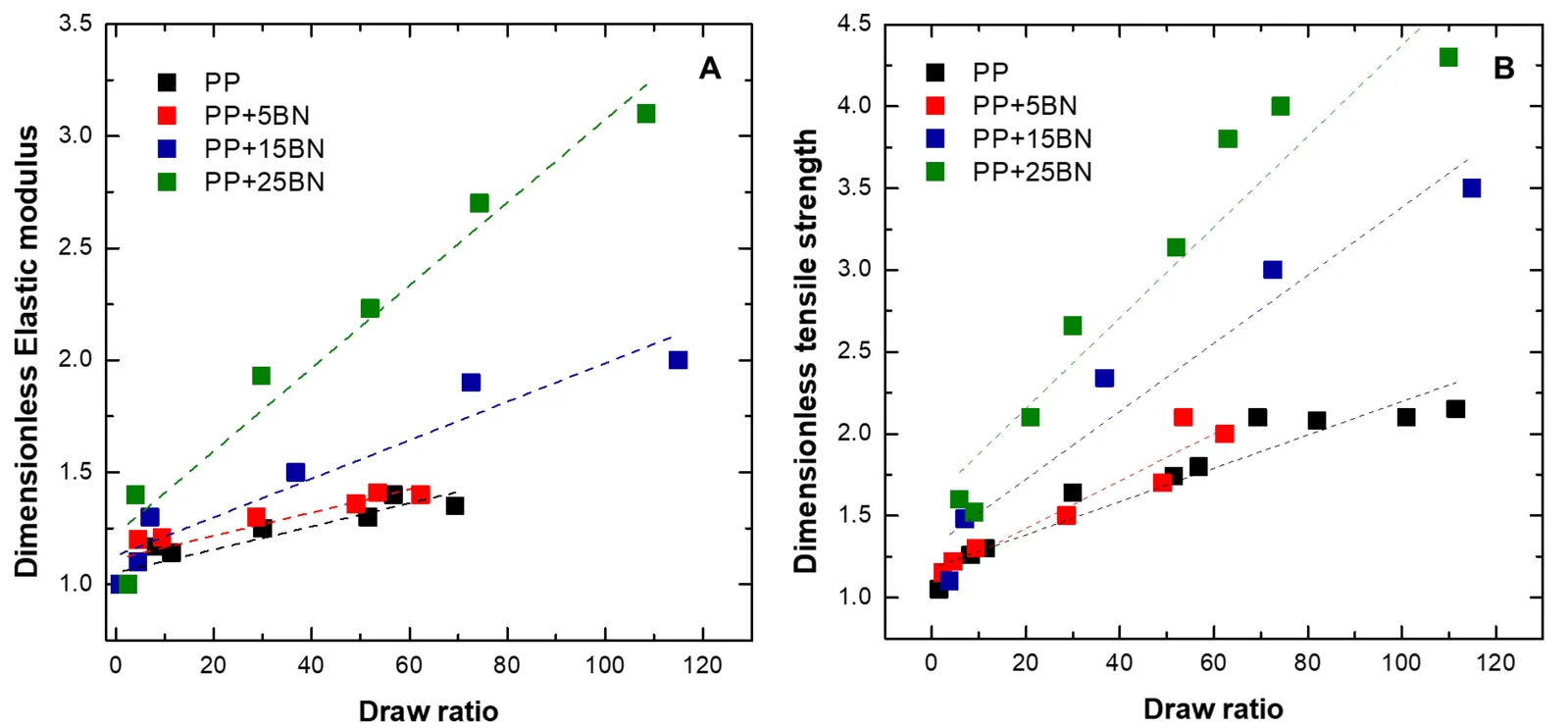
Dimensionless elastic modulus (**A**) and tensile strength (**B**) as a function of draw ratio for all investigated samples.

**Figure 5:**
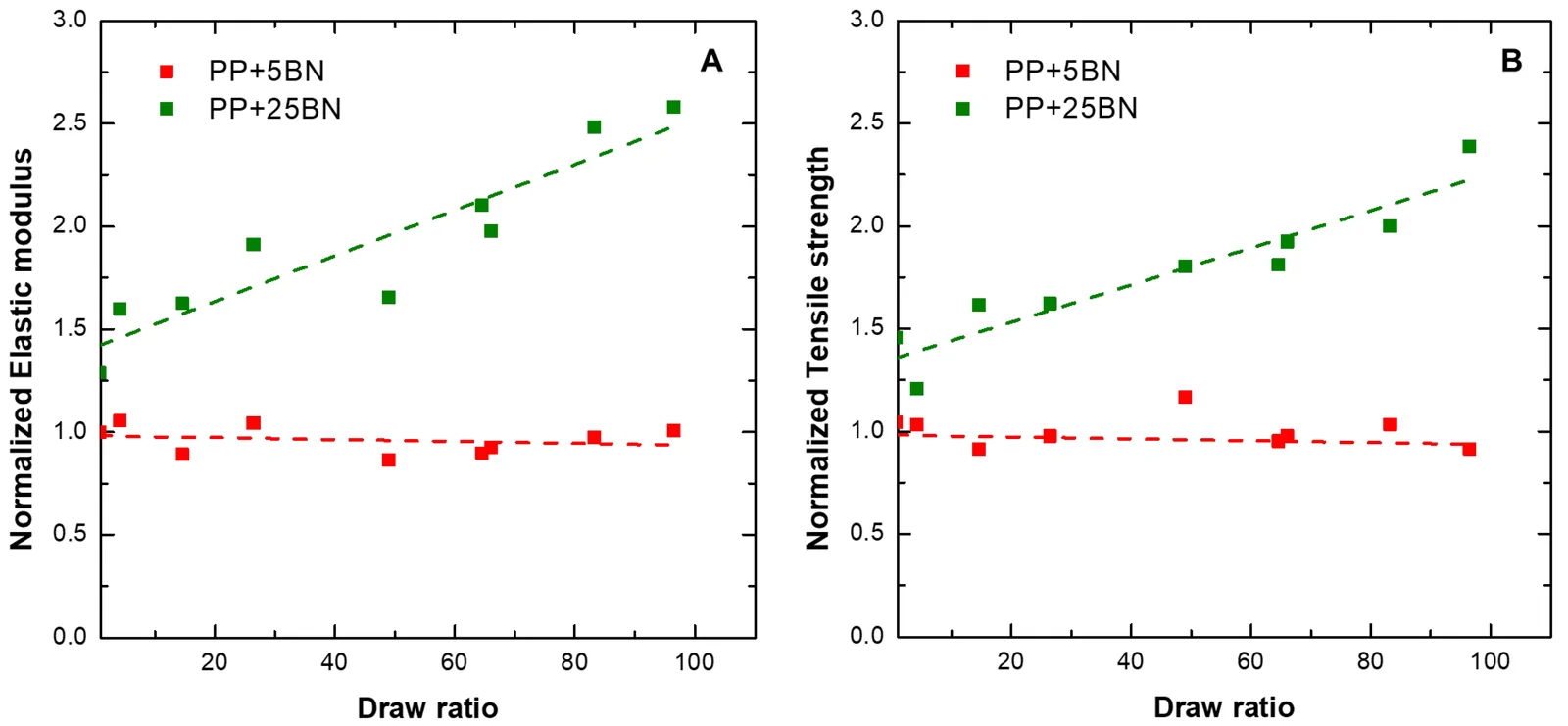
Normalized elastic modulus (**A**) and tensile strength (**B**) curves as a function of draw ratio for BN-based systems containing 5 and 25 wt% of nanofillers.

**Figure 6:**
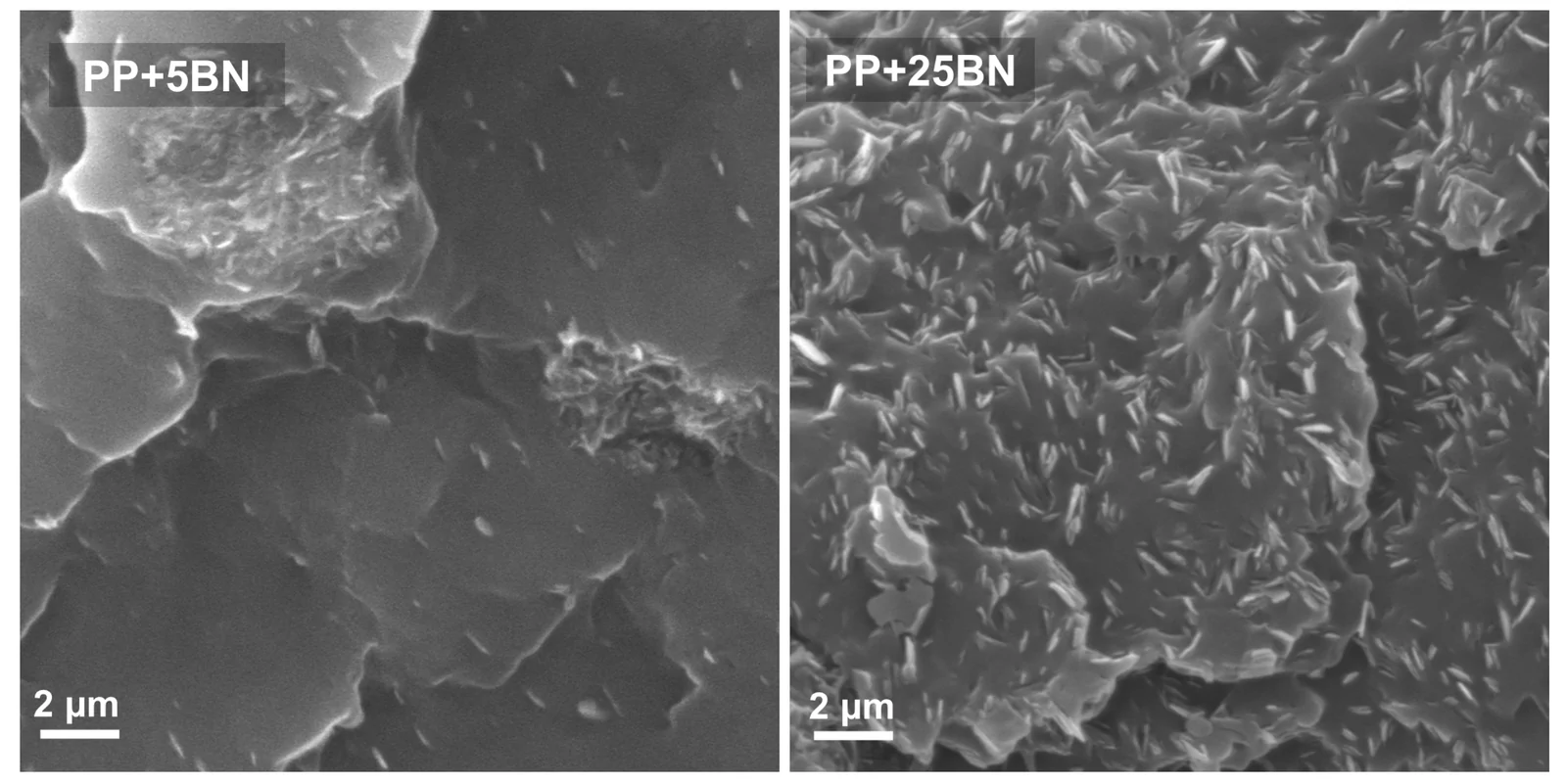
SEM micrographs of anisotropic PP composites (fibers) containing 5 and 25 wt% BN (DR = 80).

**Figure 7:**
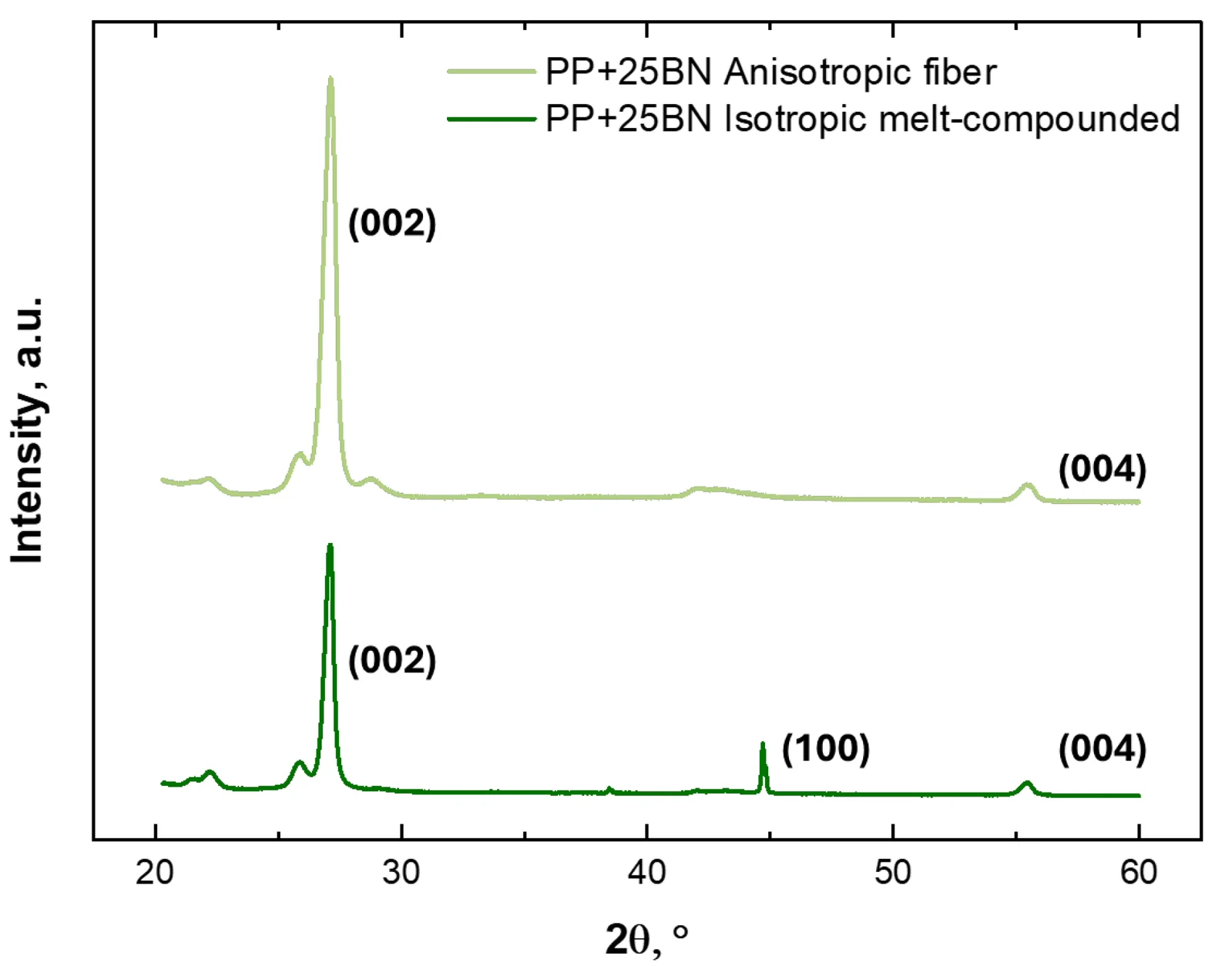
XRD patterns of isotropic (melt-compounded) and anisotropic (fiber at DR = 80) PP+25BN samples.

**Figure 8:**
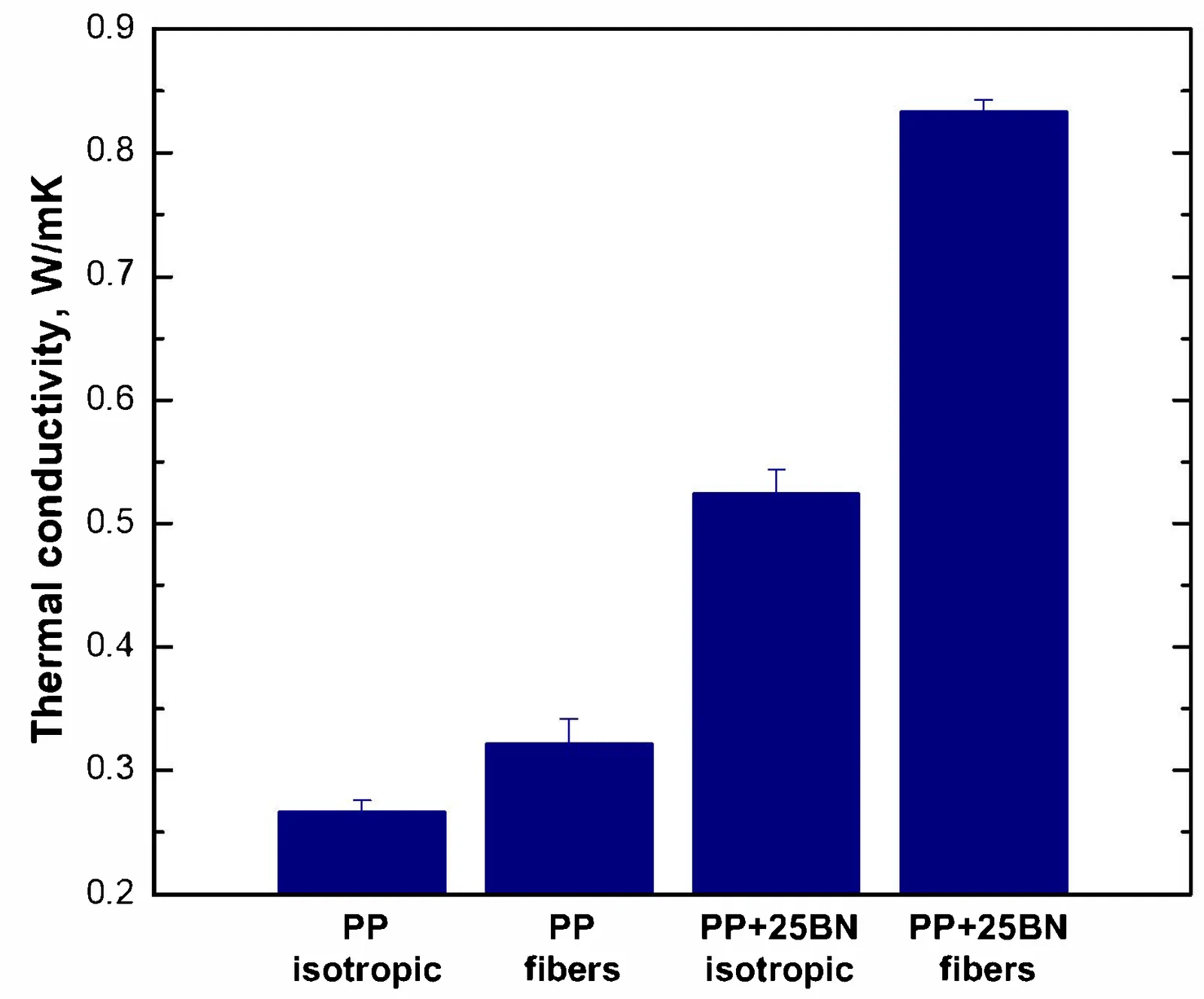
Thermal conductivity values for unfilled PP and PP+25BN fibers (DR = 80). The values of thermal conductivity for the corresponding isotropic (melt-compounded) samples are also depicted.

**Table 1:** Main results of DSC and tensile characterizations, and thermal conductivity values for all investigated materials.

	T_m_ °C	Xc %	E MPa	TS MPa	EB %	Thermal Conductivity W/mK
PP	166.2	45	854 ± 81	17.5 ± 2.1	32.6 ± 0.6	0.266 ± 0.01
PP+5BN	166.3	47	1193 ± 34	19.4 ± 0.4	21.3 ± 0.8	0.325 ± 0.015
PP+15BN	166.3	48	1406 ± 43	23.7 ± 1.5	18.9 ± 0.6	0.384 ± 0.017
PP+25BN	165.9	49	1660 ± 35	27.8 ± 0.7	14.2 ± 0.2	0.524 ± 0.02

## Data Availability

The raw data supporting the conclusions of this article will be made available by the authors on request.
